# Waiting time in diagnosis and extirpative surgery and association with survival and stage progression in upper tract urothelial carcinomas

**DOI:** 10.1002/bco2.70093

**Published:** 2025-09-22

**Authors:** Fredrik Liedberg, Oskar Hagberg, Christel Häggström, Firas Aljabery, Truls Gårdmark, Staffan Jahnson, Tomas Jerlström, Viveka Ströck, Karin Söderkvist, Anders Ullén, Lars Holmberg, Johannes Bobjer

**Affiliations:** ^1^ Department of Urology Skåne University Hospital Malmö Sweden; ^2^ Institution of Translational Medicine Lund University Malmö Sweden; ^3^ Department of Surgical Sciences Uppsala University Uppsala Sweden; ^4^ Northern Registry Centre, Department of Diagnostics and Intervention Umeå University Sweden; ^5^ Department of Clinical and Experimental Medicine, Division of Urology Linköping University Linköping Sweden; ^6^ Department of Clinical Sciences, Danderyd Hospital Karolinska Institute Stockholm Sweden; ^7^ Department of Urology, School of Medical Sciences, Faculty of Medicine and Health Örebro University Örebro Sweden; ^8^ Department of Urology, Sahlgrenska University Hospital and Institute of Clinical Sciences, Sahlgrenska Academy University of Gothenburg Sweden; ^9^ Department of Diagnostics and Intervention Umeå University Umeå Sweden; ^10^ Department of Oncology‐Pathology Karolinska Institutet Stockholm Sweden; ^11^ Department of Pelvic Cancer, Genitourinary Oncology and Urology unit Karolinska University Hospital Stockholm Sweden; ^12^ School of Cancer and Pharmaceutical Sciences King's College London London UK

**Keywords:** diagnostic delay, radical nephroureterectomy, segmental ureterectomy, total delay, treatment delay, upper tract urothelial carcinoma

## Abstract

**Objectives:**

To investigate the association between waiting time and outcomes in patients with upper tract urothelial carcinomas (UTUC).

**Patients and methods:**

We studied a population‐based cohort of 858 patients in BladderBaSe 2.0 subjected to extirpative surgery for UTUC 2015–2019 in Sweden. Diagnostic waiting time (from referral to diagnosis, reference <1 week), treatment waiting time (from diagnosis to surgery, reference <5 weeks) and total waiting time (reference <10 weeks) were investigated in relation to disease‐specific (DSS) and overall survival (OS) by multivariable Cox regression models. To further explore these associations, stage progression from preoperatively recorded clinical tumour stage to pathological tumour stage in the extirpated specimen was assessed by logistic regression.

**Results:**

Total waiting time was not associated with DSS, OS or stage progression. A diagnostic waiting time between 1 and 4 weeks was associated with better DSS (HR 0.57 [95% CI 0.35–0.94]) and OS (HR 0.60 [95% CI 0.41–0.87]). In the strata of patients with UTUC in the renal pelvis, a diagnostic waiting time > 4 weeks was associated with stage progression (OR 2.44 [95% CI 1.00–5.95]), and in patients with UTUC in the ureter, a treatment waiting time between 5 and 10 weeks was associated to worse DSS (HR 2.85 (95% CI 1.03–7.89).

**Conclusions:**

In general, shorter care pathways were linked to beneficial survival estimates, yet some estimates may be influenced by selection bias due to prioritizing short waiting times for patients with advanced and/or overt symptomatic tumours. Stage progression with increased waiting time may indicate an underlying causal mechanism.

## 1 INTRODUCTION

Since early reports highlighted the negative impact of treatment delays on outcomes in bladder urothelial carcinoma,^1^ subsequent studies have reinforced this association.^2^ However, evidence regarding upper tract urothelial carcinoma (UTUC) remains limited and inconsistent. Some studies found no link between treatment delays and survival,^3^, ^4^, ^5^ while others reported worse outcomes with prolonged waiting times.^6^, ^7^, ^8^ Adverse effects of delays have been particularly noted in patients with hydronephrosis^9^ and ureteric tumours.^10^ Interpretation of these findings is complicated by inconsistent definitions of waiting times and the lack of distinction between diagnostic delay (referral to diagnosis) and treatment delay (diagnosis to surgery).

This study aimed to assess the impact of waiting times on survival and stage progression in UTUC patients undergoing extirpative surgery between 2015 and 2019, using data from the Bladder Cancer Data Base Sweden (BladderBaSe 2.0),^11^ which includes all cases from the Swedish National Registry of Urinary Bladder Cancer (SNRUBC). Standardized care pathways (SCP) for patients with macroscopic haematuria were introduced in late 2015 in Sweden; thus as an additional analysis, we evaluated the effect of SCP introduction on waiting times.

## 2 PATIENTS AND METHODS

### 2.1 Study population

We included patients diagnosed with UTUC from 2015 to 2019 in BladderBaSe 2.0. Data on referral, diagnosis, surgery dates, tumour characteristics and treatment were obtained from the SNRUBC. Socioeconomic status was approximated using education level from the Longitudinal Integration Database for Health Insurance and Labour Market Studies (LISA), and comorbidity was assessed via the Drug Comorbidity Index (DCI) using the Prescribed Drug Register.^12^ Swedish national guidelines recommend multidisciplinary tumour board (MDT) discussions for all UTUC cases, following EAU guidelines,^13^, ^14^ though diagnostic practices vary across institutions.^15^

**FIGURE 1:**
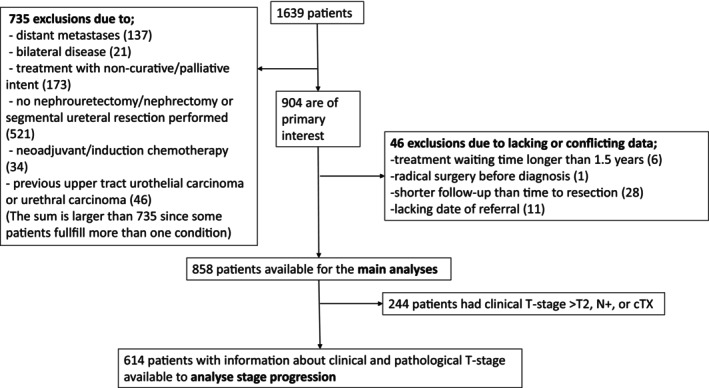
Flow‐chart of the study population. *The sum is larger than 735 since some patients fulfil more than one condition.

Exclusion criteria were: primary distant metastases, bilateral UTUC, non‐curative treatment, endourologic procedures (without nephroureterectomy/nephrectomy or segmental ureterectomy), preoperative chemotherapy, prior UTUC or urethral carcinoma, excessive waiting time (>1.5 years), surgery before diagnosis, follow‐up shorter than time to treatment or missing referral date. Patients with diagnosis preceding referral were also excluded. For stage progression analysis, we excluded cases with clinical stage >T2, clinical lymph node metastasis, or unknown tumour stage (Tx) (Figure 1).

### 2.2 Exposure and outcomes

*Total waiting time* was defined as the interval from either referral or diagnosis (whichever occurred first) to the date of extirpative surgery—radical nephroureterectomy (RNU), nephrectomy or segmental ureterectomy. Diagnosis was based on clinical (cytology/radiology) and/or pathological findings. Waiting time was further divided into *diagnostic waiting time* (referral to diagnosis) and *treatment waiting time* (diagnosis to surgery). Thus, the latter exposures have potential considerable overlap with total waiting time.

Data on disease‐specific survival (DSS) and overall survival (OS) were obtained from the Swedish Cause of Death Register and the Register of Total Population and Population Changes. DSS was defined as death due to urothelial carcinoma of the renal pelvis (ICD‐10 C65), ureter (C66), bladder (C67) or urethra (C68). Stage progression was assessed in patients with clinically organ‐confined disease (≤cT2N0M0) and defined as an increase in tumour stage from clinical to pathological staging, categorized as:

1. Ta/Tis/T1 and N0
2. T2 and N0
3. T3/T4 or any T with N1–2 or M+

### 2.3 Statistical analyses

Follow‐up for survival analyses began at surgery and ended at the earliest of death (from urothelial cancer or any cause), migration, or 31 December 2019. Waiting times were categorized into tertiles closest to whole weeks, with the lowest tertile as reference (0–1 week for diagnostic‐, 0–5 weeks for treatment‐ and 0–10 weeks for total waiting time). Patients with a diagnosis preceding referral were excluded from analyses.

We used multivariable Cox regression models for the association of waiting times and DSS/OS to control for potential selection of patients to longer or shorter waiting times due to factors that may impact survival. The models were adjusted for age, sex, comorbidity (three categories),^12^ education level (low/middle/high), prior bladder cancer, tumour location (renal pelvis/ureter), clinical tumour and nodal stage, tumour grade, and adjuvant chemotherapy. Stratified analyses were conducted for renal pelvis and ureteric tumours, based on the assumption that renal pelvic tumours are less likely to cause hydronephrosis and thus less influenced by diagnostic delays. A subgroup analysis was also performed for patients without prior bladder cancer, assuming different diagnostic and survival dynamics.

To explore whether tumour progression mediates the relationship between waiting time and survival, logistic regression was used to assess the association between waiting time and stage progression, adjusting for age, sex, tumour grade, comorbidity, prior bladder cancer, and tumour location. Analyses were repeated in subgroups by tumour location and prior bladder cancer status. Comparison of waiting time in patients treated within or outside a SCP was performed by Wilcoxon rank sum test. All analyses were conducted using R version 4.1.1.

## 3 RESULTS

After applying exclusion criteria, 858 patients who underwent extirpative surgery were included in the survival analysis, and 610 were eligible for stage progression analysis (Figure 1).

Among the cohort, 542 patients (63%) had tumours in the renal pelvis and 316 (37%) in the ureter. In addition to mandatory cystoscopy and voided urine cytology, 423 patients (49%) were diagnosed by imaging alone, 59 (6.9%) underwent selective upper tract urine sampling and 376 (44%) had ureteroscopic evaluation with or without biopsy. Most patients (712; 83%) received nephroureterectomy, while 47 (6%) underwent nephrectomy and 99 (12%) segmental ureteral resection. Patient, tumour and treatment characteristics were generally balanced across tertiles of total waiting time, with minor differences in prior bladder cancer, tumour location, nodal stage, comorbidity index and education level (Table 1).

**TABLE 1:** Baseline patient, tumour and treatment characteristics of the studied population with patients diagnosed with upper tract urothelial carcinoma (UTUC) and treated with extirpative surgery between 2015 and 2019 in BladderBaSe 2.0.

Total waiting time	Weeks	0–10	10–15	15‐	Total
	n	349	229	280	858
***Age (years)***	*Median*	72	74	74	73
	*IQR*	67–77	69–80	70–80	68–79
***Gender***	*Male*	210 (60%)	146 (64%)	175 (63%)	531 (62%)
	*Female*	139 (40%)	83 (36%)	105 (38%)	327 (38%)
***Side***	*Right*	168 (48%)	121 (53%)	137 (49%)	426 (50%)
	*Left*	181 (52%)	108 (47%)	143 (51%)	432 (50%)
***Previous bladder cancer***	*Yes*	58 (17%)	57 (25%)	89 (32%)	204 (24%)
	*No*	291 (83%)	172 (75%)	191 (68%)	654 (76%)
***Tumour location*** ^***a***^	*Renal pelvis*	247 (71%)	143 (62%)	152 (54%)	542 (63%)
	*Ureter*	102 (29%)	86 (38%)	128 (46%)	316 (37%)
***Clinical tumour stage***	*Ta*	127 (36%)	116 (51%)	135 (48%)	378 (44%)
	*Tis*	7 (2.0%)	6 (2.6%)	9 (3.2%)	22 (2.6%)
	*T1*	62 (18%)	26 (11%)	40 (14%)	128 (15%)
	*T2*	40 (12%)	23 (10%)	24 (8.6%)	87 (10%)
	*T3*	83 (24%)	41 (18%)	50 (18%)	174 (20%)
	*T4*	17 (4.9%)	8 (3.5%)	2 (0.7%)	27 (3.1%)
	*TX*	13 (3.7%)	9 (3.9%)	20 (7.1%)	42 (4.9%)
***Clinical grade*** ***(WHO 1999)***	*G1/LMP*	51 (15%)	36 (16%)	50 (18%)	137 (16%)
	*G2*	131 (48%)	98 (43%)	92 (33%)	321 (37%)
	*G3*	145 (32%)	85 (37%)	126 (45%)	356 (42%)
	*GX*	22 (6.3%)	10 (4.4%)	12 (4.3%)	44 (5.1%)
***Clinical nodal stage***	*N0*	290 (83%)	182 (80%)	186 (66%)	658 (77%)
	*N1*	9 (2.6%)	6 (2.6%)	8 (2.9%)	23 (2.7%)
	*N2*	8 (2.3%)	4 (1.7%)	4 (1.4%)	16 (1.9%)
	*NX*	42 (12%)	37 (16%)	82 (29%)	161 (19%)
***Type of surgery***	*Nephroureterectomy*	296 (85%)	188 (82%)	228 (81%)	712 (83%)
	*Segmental ureterectomy*	29 (8.3%)	29 (13%)	41 (15%)	99 (12%)
	*Nephrectomy*	24 (6.9%)	12 (5.2%)	11 (3.9%)	47 (5.5%)
***Drug Comorbidity Index (DCI)***	*0–0.75*	131 (38%)	87 (38%)	81 (29%)	299 (35%)
	*0.75–2.00*	107 (31%)	71 (31%)	105 (38%)	283 (33%)
	*2.00 and above*	111 (32%)	71 (31%)	94 (34%)	276 (32%)
***Education level***	*Low*	111 (33%)	84 (38%)	106 (39%)	301 (36%)
	*Middle*	147 (43%)	93 (42%)	120 (44%)	360 (43%)
	*High*	84 (25%)	47 (21%)	49 (18%)	180 (21%)
	*Missing*	7	5	5	17

^a^ A total of 31 patients with tumour location/s in both renal pelvis and ureter were included in the renal pelvis group. IQR = Interquartile range, LMP = low malignant potential.

The median total waiting time from referral to surgery was 82 days (IQR 52–122), with a median diagnostic waiting time of 16 days (IQR 6–40) and treatment waiting time of 54 days (IQR 32–87). Over a median follow‐up of 2.13 years, 185 patients (22%) died, including 109 (13%) from urothelial cancer. Stage progression occurred in 98 patients (16%).

### 3.1 Total waiting time

No significant differences in DSS or OS were observed across tertiles of total waiting time (Table 2). Similarly, no association was found between total waiting time and stage progression (Table 3).

**TABLE 2:** Hazard ratios (HRs) and 95% confidence intervals (CI) based on multivariable Cox regression on total waiting time, diagnostic waiting time and treatment waiting time and the association to disease‐specific (DSS) and overall survival (OS).

UTUC study population			DSS		OS	
	weeks	N	HR	95% CI	HR	95% CI
Total waiting time	0–10	349	1	‐	1	‐
	10–15	229	0.97	0.59–1.61	1.07	0.74–1.56
	15‐	280	1.02	0.64–1.63	1.10	0.77–1.57
Diagnostic waiting time	0–1	270	1	‐	1	‐
	1–4	293	0.57*	0.35–0.94	0.60**	0.41–0.87
	4‐	295	0.83	0.52–1.32	0.82	0.58–1.16
Treatment waiting time	0–5	255	1	‐	1	‐
	5–10	281	0.91	0.55–1.49	0.99	0.68–1.44
	10‐	322	1.10	0.69–1.78	1.15	0.80–1.67

UTUC in renal pelvis			DSS		OS	
	weeks	n	HR	95% CI	HR	95% CI
Total waiting time	0–10	247	1	‐	1	‐
	10–15	143	0.99	0.52–1.89	1.30	0.81–2.08
	15‐	152	1.43	0.82–2.49	1.48	0.96–2.29
Diagnostic waiting time	0–1	164	1	‐	1	‐
	1–4	198	0.53*	0.28–1.00	0.51**	0.32–0.82
	4‐	180	1.06	0.59–1.92	0.94	0.60–1.47
Treatment waiting time	0–5	181	1	‐	1	‐
	5–10	183	0.53	0.28–1.00	0.80	0.50–1.27
	10‐	178	1.06	0.60–1.86	1.24	0.80–1.94

UTUC in ureter			DSS		OS	
	weeks	n	HR	95% CI	HR	95% CI
Total waiting time	0–10	102	1	‐	1	‐
	10–15	86	0.96	0.41–2.22	0.76	0.40–1.44
	15‐	128	0.55	0.24–1.26	0.62	0.34–1.14
Diagnostic waiting time	0–1	106	1	‐	1	‐
	1–4	95	0.69	0.28–1.68	0.79	0.42–1.49
	4‐	115	0.60	0.27–1.34	0.65	0.36–1.18
Treatment waiting time	0–5	74	1	‐	1	‐
	5–10	98	2.85*	1.03–7.89	1.51	0.77–3.00
	10‐	144	1.56	0.56–4.37	1.01	0.51–2.02

* p < 0.05,

** p < 0.01.

HRs adjusted for age, gender, comorbidity, education level, previous bladder cancer, tumour location (for the full UTUC study population only), clinical tumour stage and clinical nodal stage groups, clinical grade and postoperative treatment with adjuvant chemotherapy.

**TABLE 3:** Odds ratios (ORs) and 95% confidence intervals (CI) based on a logistic regression model investigating the association between total waiting time, diagnostic waiting time and treatment waiting time and stage progression in the resection specimen compared to the preoperative clinical stage group.

	UTUC study population				UTUC in renal pelvis			UTUC in ureter		
	Stage progression				Stage progression			Stage progression		
	weeks	n	OR	95% CI	n	OR	95% CI	n	OR	95% CI
Total waiting time	0–10	235	1	‐	154	1	‐	81	1	‐
	10–15	171	1.27	0.72–2.24	98	1.15	0.50–2.63	73	1.33	0.59–2.98
	15‐	208	1.20	0.70–2.07	99	1.44	0.65–3.18	109	0.98	0.45–2.12
Diagnostic waiting time	0–1	192	1	‐	103	1	‐	89	1	‐
	1–4	212	0.83	0.46–1.49	134	1.12	0.44–2.86	78	0.71	0.32–1.58
	4‐	210	1.50	0.88.2.56	114	2.44*	1.00‐5.95	96	1.07	0.53–2.17
Treatment waiting time	0–5	174	1	‐	114	1	‐	60	1	‐
	5–10	199	1.15	0.64–2.07	119	0.77	0.34–1.75	80	2.02	0.79–5.16
	10‐	241	1.12	0.64–1.98	118	0.77	0.35–1.72	123	1.74	0.71–4.24

* p < 0.05, OR = Odds Ratio, adjusted for age, gender, clinical grade, comorbidity, previous bladder cancer and tumour location (for the full UTUC study population only). Stage progression was assessed in patients with clinically organ‐confined disease (cT2N0M0 or less) and defined as an increase in tumour stage group from preoperatively recorded clinical tumour stage to pathological tumour stage in the extirpated specimen ordered as follows: 1) Ta/Tis/T1 and N0, 2) T2 and N0, 3) T3/T4 or any T and N1–2 or M+.

### 3.2 Diagnostic waiting time

A diagnostic waiting time of 1–4 weeks was associated with improved DSS (HR 0.57, 95% CI 0.35–0.94) and OS (HR 0.60, 95% CI 0.41–0.87) (Table 2). This association was evident in patients with renal pelvic tumours but not in those with ureteric tumours. A diagnostic delay of more than 4 weeks was linked to increased stage progression in renal pelvic UTUC (OR 2.44, 95% CI 1.0–5.95) (Table 3).

### 3.3 Treatment waiting time

Overall, treatment waiting time was not significantly associated with DSS or OS (Table 2). However, among patients with ureteric tumours, a delay of 5–10 weeks was associated with worse DSS (HR 2.85, 95% CI 1.03–7.89). No significant association was found between treatment waiting time and stage progression (Table 3).

Subgroup analysis of patients without prior bladder cancer yielded similar results (data not shown). Moreover, the median total waiting time for patients subjected to SCP was shorter compared to those managed outside of SCP (78 (IQR 50–112) vs 86 (IQR 54–130) days, respectively (p = 0.04)), whereas diagnostic waiting time and treatment waiting time were similar regardless of SCP or not (15 (IQR 6–36) vs 19 (IQR 5–46) and 52 (IQR 32–87) vs 55 (IQR 31–86) days, respectively).

## 4 DISCUSSION

In patients with UTUC, we found a pattern with longer waiting time and association with unfavourable outcomes. Patients with UTUC in the ureter and a treatment waiting time above five weeks were associated with worse DSS, and patients with UTUC in the renal pelvis and a diagnostic waiting time of more than four weeks had an association with risk of stage progression. However, an intermediate diagnostic waiting time between 1 and 4 weeks was counterintuitively associated with improved DSS and OS compared to waiting times <1 week.

Our finding that the strata of patients with UTUC in the ureter are more sensitive to treatment waiting time beyond five weeks compared to UTUC in the renal pelvis regarding DSS aligns with previous findings.^10^ One tentative reason for ureteric tumours being more prone to progress during treatment waiting time is that the ureter compared to the renal pelvis has a thinner layer of ureteral adventitia enriched with blood and lymphatic vessels, promoting lymphatic and haematogenous spread.^16^ Additionally, ureteric tumours lack renal parenchyma as a barrier for tumour spread, allowing for wider surgical resection margins for UTUC in the renal pelvis.^17^ Additionally, ureteric tumours more frequently cause hydronephrosis, a known negative prognostic factor,^18^ which may contribute to tumour progression through mechanical and vascular mechanisms.^19^

Studies reporting no survival impact from treatment delays often included fewer ureteric tumours with limited statistical precision to detect modest but clinically relevant survival differences.^3^, ^4^, ^5^ In contrast, studies with larger ureteric tumour cohorts, including ours (n = 316), have demonstrated significant associations.^6^, ^7^ Our findings support recommendations to limit treatment delays for ureteric UTUC to under 35 days.^10^

For patients with UTUC in the renal pelvis a diagnostic waiting time more than four weeks was associated with stage progression (Table 3). This finding strengthens the general findings since tumour progression, even in shorter time frames as in the current study, represent a possible causal mechanism for the association between waiting time and survival outcomes.

The observed survival benefit for intermediate diagnostic waiting times (1–4 weeks) and the lack of association with total waiting time may reflect selection bias (Table 3). Patients with more advanced or symptomatic disease may be prioritized for earlier surgery, skewing survival outcomes despite statistical adjustments. These selection mechanisms are likely to be more pronounced in the context of treatment waiting times, as clinicians may be influenced by imaging results that reflect disease severity. Similar mechanisms have been described in bladder cancer.^20^

The Standardized Care Pathway (SCP), introduced in Sweden in 2015 for patients with macroscopic haematuria, established a maximum total waiting time of 37 days from referral to radical cystectomy for bladder cancer patients.^21^ Although no equivalent limit exists for UTUC in Swedish guidelines, our findings indicate shorter total waiting times for UTUC patients treated within the SCP compared to those outside it. Recent efforts, such as those by Parmar et al.,^22^ have demonstrated that a multidisciplinary approach with dedicated diagnostic and surgical resources can reduce treatment delays and decrease the usage of diagnostic ureteroscopy. These initiatives, including SCP, likely contribute to improved survival by streamlining the patient pathway.

This is the first nationwide study in UTUC to incorporate detailed data on patient timelines, tumour characteristics and treatment, enabling robust control for confounding and enhancing clinical relevance. However, we could not control for symptoms prompting clinical prioritization. We also lack data on the presence of hydronephrosis^9^, ^23^ multifocality,^16^ and tumour size,^24^ precluding risk stratification per EAU guidelines. Some patients may have progressed to non‐curable disease due to delays, though we lack data on their proportion. To reduce bias, we excluded those treated with non‐curative intent or with metastases, which may underestimate the impact of waiting time. Also, we acknowledge the known difficulties in clinical tumour staging in lower tumour stages, which is the baseline for the logistic model assessing stage progression.^25^ The heterogenous results derived from this assessment may be due to the aforementioned difficulties in staging as well as a sometimes complex clinical work‐up in UTUC impacting lead times in a non‐systematic fashion. Lastly, despite including more than 800 individuals, which enables tumour location‐specific subgroup analysis, the numbers of patients and events are limited in some cells entailing a limited statistical precision and some modest but clinically relevant associations may go undetected.

## 5 CONCLUSIONS

Survival estimates in the present study point to benefits from shorter care pathways, yet some estimates may be influenced by selection bias due to prioritizing short waiting times for patients with advanced and/or overt symptomatic tumours. The findings add support to keep diagnostic waiting time shorter than four weeks for patients with UTUC in the renal pelvis and treatment waiting time below five weeks in patients with UTUC in the ureter. The association of stage progression to waiting time represent a plausible causal mechanism. Since most of these patients present with macroscopic haematuria, our results support the implementation of timely diagnosis for all haematuria patients, as well as performing timely surgery when a patient is diagnosed with UTUC, especially when located in the ureter.

## AUTHOR CONTRIBUTIONS

**Fredrik Liedberg:** Conceptualization; data curation; funding acquisition; investigation; methodology; project administration; writing—original draft. **Oskar Hagberg:** Formal analysis; writing—review and editing. **Christel Häggström:** Formal analysis; writing—review and editing. **Firas Aljabery:** Investigation; writing—review and editing. **Truls Gårdmark:** Investigation; writing—review and editing. **Staffan Jahnson:** Investigation; writing—review and editing. **Tomas Jerlström:** Investigation; writing—review and editing. **Viveka Ströck:** Investigation; writing—review and editing. **Karin Söderkvist:** Investigation; writing—review and editing. **Anders Ullén:** Investigation; writing—review and editing. **Lars Holmberg:** Conceptualization; data curation; funding acquisition; investigation; writing—review and editing. **Johannes Bobjer:** Formal analysis; investigation; methodology; writing—original draft.

## CONFLICT OF INTEREST STATEMENT

The authors have no relevant financial or non‐financial interests to disclose.
